# Morphology and morphometry of pulmonary veins and the left atrium in multi-slice computed tomography

**DOI:** 10.1007/s00276-019-02210-1

**Published:** 2019-03-02

**Authors:** Mateusz Polaczek, Pawel Szaro, Inga Baranska, Barbara Burakowska, Bogdan Ciszek

**Affiliations:** 1 Department of Descriptive and Clinical Anatomy, Medical University of Warsaw, Warsaw, Poland; 2 Third Department of Lung Diseases and Oncology, National Tuberculosis and Lung Diseases Research Institute, Plocka 26, 01138 Warsaw, Poland; 3 Department of Radiology, Sahlgrenska University of Gothenburg, Gothenburg, Sweden; 4 Department of Radiology, National Tuberculosis and Lung Diseases Research Institute, Warsaw, Poland

**Keywords:** Pulmonary veins, Left atrium, Anatomy, Pulmonary vein ostium, Computed tomography, Atrial fibrillation

## Abstract

**Purpose:**

Pulmonary veins are involved in pathogenesis and treatment of atrial fibrillation and structures at risk during thoracic surgeries. There is lack of data regarding pulmonary vein morphology and morphometry in normal population.

**Methods:**

The study was conducted using 135 chest computed tomography studies with intra-venous iodine contrast injection. The study population contained 86 females and 49 males, mean age was 60. 13 had atrial fibrillation. The studies were analyzed using radiological workstation.

**Results:**

Mean dimensions of the left atrium: transverse 52 mm, coronal 49 mm, and sagittal 35 mm. The mean volume of the left atrium was 93 cm^3^. The mean volume of the left atrium in patients with atrial fibrillation was 176 cm^3^. The sagittal dimension and the volume of the left atrium were correlated with age, *r* = 0.43 and *r* = 0.42, respectively. Surface area of the left inferior pulmonary vein ostium was 136 mm^2^, significantly less than the surface area of other ostia of pulmonary veins. The mean distance between two pulmonary veins was 5.42 mm on the right and 4.02 mm on the left side. 13 types of pulmonary veins outflow patterns were described on the right side and 5 types on the left side. 66.7% of right pulmonary veins and 82% of the left pulmonary veins emptied into the left atrium with two venous trunks on each side (the typical pattern).

**Conclusions:**

Morphological features of pulmonary veins and morphometry of the left atrium and pulmonary veins are important for clinical purposes and are in accordance with previous papers.

## Introduction

Pulmonary veins (PVs) and the left atrium (LA) are central in pathogenesis of atrial fibrillation (AF) [9, 26]. This supraventricular tachyarrhythmia not only propagates from the proximal part of PVs (ectopic beats starts mainly in upper PVs) [12], but is also the main cause of morphometric changes of LA and the proximal part of PVs [15]. There are suggestions that not only histological features, but also gross morphology of PVs ostia, can predict the occurrence of atrial fibrillation [19, 22]. This is why PVs and LA anatomy were investigated many times in populations of patients with AF [5, 7, 17].

The modern era of minimally invasive thoracic procedures such as video-assisted thoracic surgery (VATS) for lung cancer resection puts new challenges for surgeons. The atypical venous anatomy during lung resection was acknowledged in 3.12% of 642 analyzed cases by Polaczek et al. [27]. With VATS the vison is limited, thus it can lead to misinterpreting the vascular structures [35] and cause potential surgical complications [25]. This is why it is so important to establish vascular anatomy prior to surgical treatment [11]. The use of multi-slice computed tomography (MSCT) with volume-rendering technique (VRT) before surgery leads to a better understanding of the vascular anatomy and facilitates guidance during resection [1].

There is missing data about morphology and morphometry of PVs, LA and the veno-atrial junction in population of patients with population-rate occurrence of AF, a group that would normally undergo surgical treatment for lung cancer.

## Materials and methods

The study was based on retrospective analysis of 135 chest computed tomography examinations with iodine contrast enhancement performed in our institution between 2011 and 2017. Study population was aged 18–84 (average age was 59.97), there were 86 females and 49 males. The clinical indication for MSCT was one of the following: unspecific changes in chest plain radiogram, history of haemoptysis, suspicion of pulmonary embolism and follow-up of peripheral pulmonary nodules of less than 10 nm in diameter; all the radiological reports stated no significant abnormalities, so the study population can be considered representative for the general population of patients. The main inclusion criterion was good contrast enhancement of PVs defined as the density over 80 Hounsfield Units (HU) of the region of interest (ROI) in the right inferior pulmonary vein (RIPV) trunk. The average ROI was 266.11 HU. The exclusion criteria contained all clinical conditions impacting pulmonary circulation and were described in details in the study protocol. The only exception involved patients with AF, who were included in this study, but constituted separate subgroup for analysis. There were 13 cases of AF included.

Examinations were performed using Sensation 16 (SIEMENS AG, Germany) 16-row scanner and Revolution GSI (GE Healthcare, USA) 64-row scanner. The reconstructions were made with 1.00 and 1.25 mm thick layers. Iodine intra-venous contrast was injected into the cephalic vein using automatic syringe.

After selection of 135 studies from the database the “raw” DICOM files were imported from a server (PACS) to Syngo.via—syngo.via Client 3.0 (SIEMENS Healthcare GmbH, Germany) workstation and processed in syngo.CT Vascular Analysis (SIEMENS Healthcare GmbH, Germany) work mode. The source material in axial planes was reconstructed into sagittal and coronal planes using multi-planar reformations (MPR). MPR was also used to reconstruct oblique planes. Vascular structures and left atrial appendage (LAA) were identified and presented using curved-planar reformations (CPR). VRT was used to create three-dimensional color-coded images, automatic and manual segmentation techniques were used to brush all structures covering pulmonary vessels.

All pulmonary veins were identified and the morphology of venous outflow was determined. After identification, the measurement of LA was made: dimensions in axial (LA_ax_), sagittal (LA_sag_), and coronal (LA_co_), and the LA volume was calculated using two separate formulas for cuboid LA_vol:c_ = LA_ax_*LA_sag_*LA_co_ and for ellipsoid LA_vol:e_ = ^4^/_3_∏*^LAax^/_2_*^LAsag^/_2_*^LAco^/_2_. Having CPR of LAA and of all identified PVs, the measurements of ostial parameters were made (minimal and maximal dimension, cross-sectional area). The ratio of minimal to maximal dimension was considered as the ostial narrowing. The length of each PV trunk was measured from the ostium to the first bifurcation. Shared oblique plane for unilateral PV was established, distance between veins and angle between long axes were measured.

All observations were saved as images in TIFF format, all measurements written in the study form. Statistical analysis was performed using Statistica v.13 (StataSoft). Numerical data are presented as mean/average and median (MED) value, standard deviation (SD), minimal (MIN) and maximal (MAX) value, 10th and 90th percentile (10p, 90p). Parametric data with normal distribution (in Shapiro–Wilk’s test) were analyzed with Student’s *t* test (*t*) and Pearson’s correlation (*r*). Parametric data without normal distribution and non-parametric data were analyzed with Mann–Whitney’s *U* test (*U, Z*), Spearman’s correlation (*R*) and Chi square (chi^2^, *df*). *P* value less than 0.05 was considered as statistically significant. The numbers were presented with two decimal places.

## Results

Left atrium axial dimension was the greatest of all, mean LA_ax_ = 51.69 mm (SD = 12.09), followed by the coronal dimension-mean LA_co_ = 48.60 mm (SD = 7.18) and sagittal-mean LA_sag_ = 35.00 (SD = 8.83). In subgroup without AF (*AF−*) LA_sag_ 90p was 41.00 mm (mean = 33.47, SD = 6.29), so it was significantly different when compared to AF positive (*AF*+) subgroup, where LA_sag_ 10p = 39.20 mm (mean = 49.36, SD 11.92), so an arbitrary value of 40 mm for LA_sag_ was a distinction to predict whether it was AF positive or negative case.

LA_vol:c_ was in average 92.72 cm^3^ (MED = 83.06, SD = 48.89) with minimal value as low as 18.55 cm^3^ and maximal as high as 290.48 cm^3^. LA_vol:e_ was accordingly lower (mean = 48.55 cm^3^, MED = 43.49, SD = 25.60). LA volume was significantly different when AF was taken into consideration, the median volume was twice higher in AF+ subgroup, compared to AF*−* subgroup, 166.56 cm^3^ and 78.95 cm^3^ for cuboid shape, respectively. Dimensions and volume of LA increased with age; linear correlations with age was proven for LA_sag_ (*r* = 0.43, *p* < 0.0001), for LA_vol:c_ (*r* = 0.42, *p* < 0.0001) and for LA_ax_ (*r* = 0.31, *p* = 0.0003). Only LA_co_ did not correlate with age (*r* = 0.17, *p* = 0.05).

The orifice of LAA is oval and flattened coronally; the narrowing of LAA was 0.62. The mean surface area of LAA orifice was 229.53 mm^2^ (SD = 117.64), with great difference between AF+ and AF*−* subgroups (403.62 and 210.83 mm^2^). With age the shape of LAA orifice changes, it gets less flattened and more round shaped: there was correlation between narrowing and age *r* = 0.27, *p* = 0.002. In AF+ subgroup, an increase in LA_vol:c_ led strongly to an increase of LAA orifice surface area (*R* = 0.74, *p* < 0.05). The length of LAA should be considered as individual feature, no relation to age or AF was found. The mean LAA length was 27.55 mm (range 6.00–55.10, SD = 7.75).

**Fig. 1:**
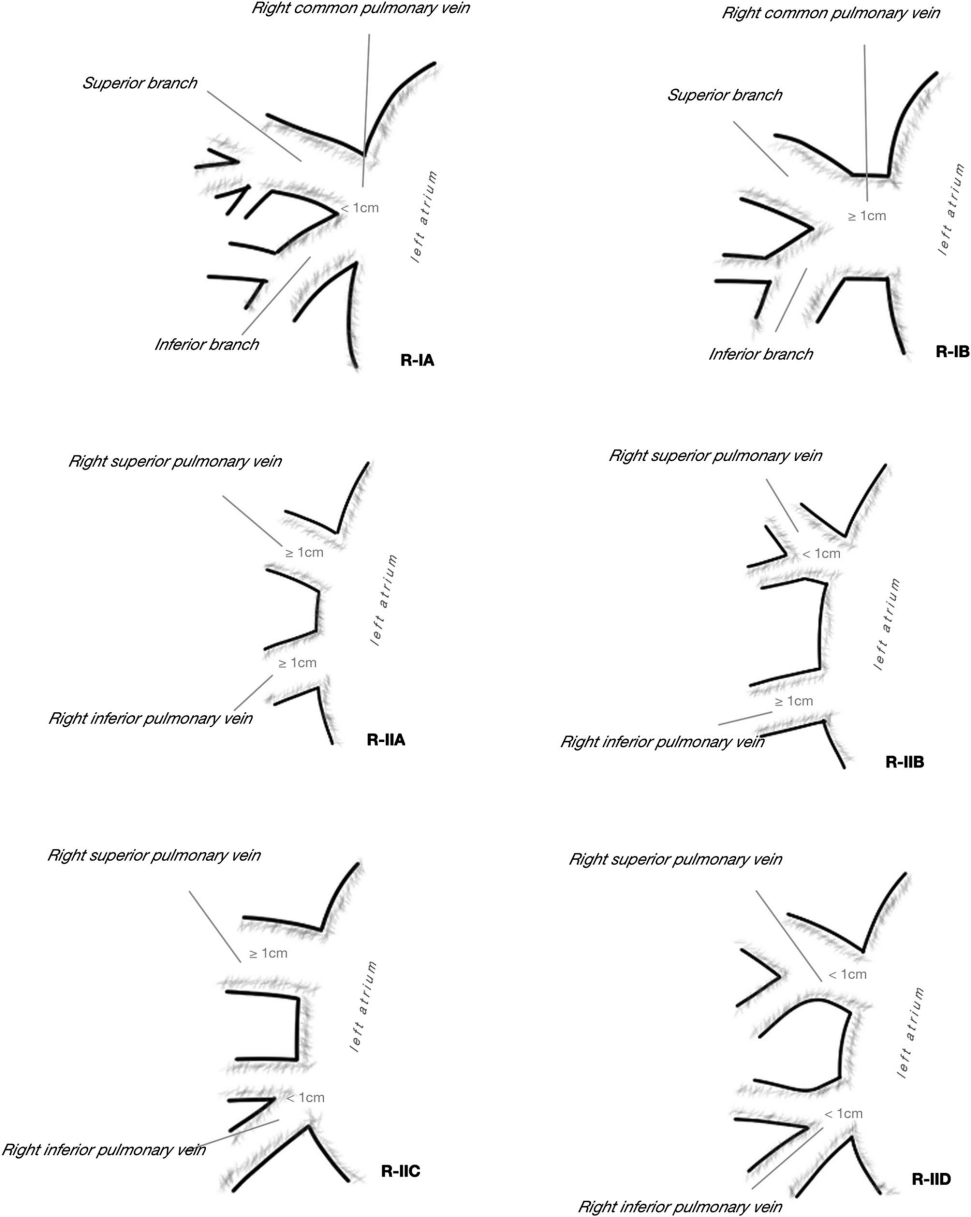

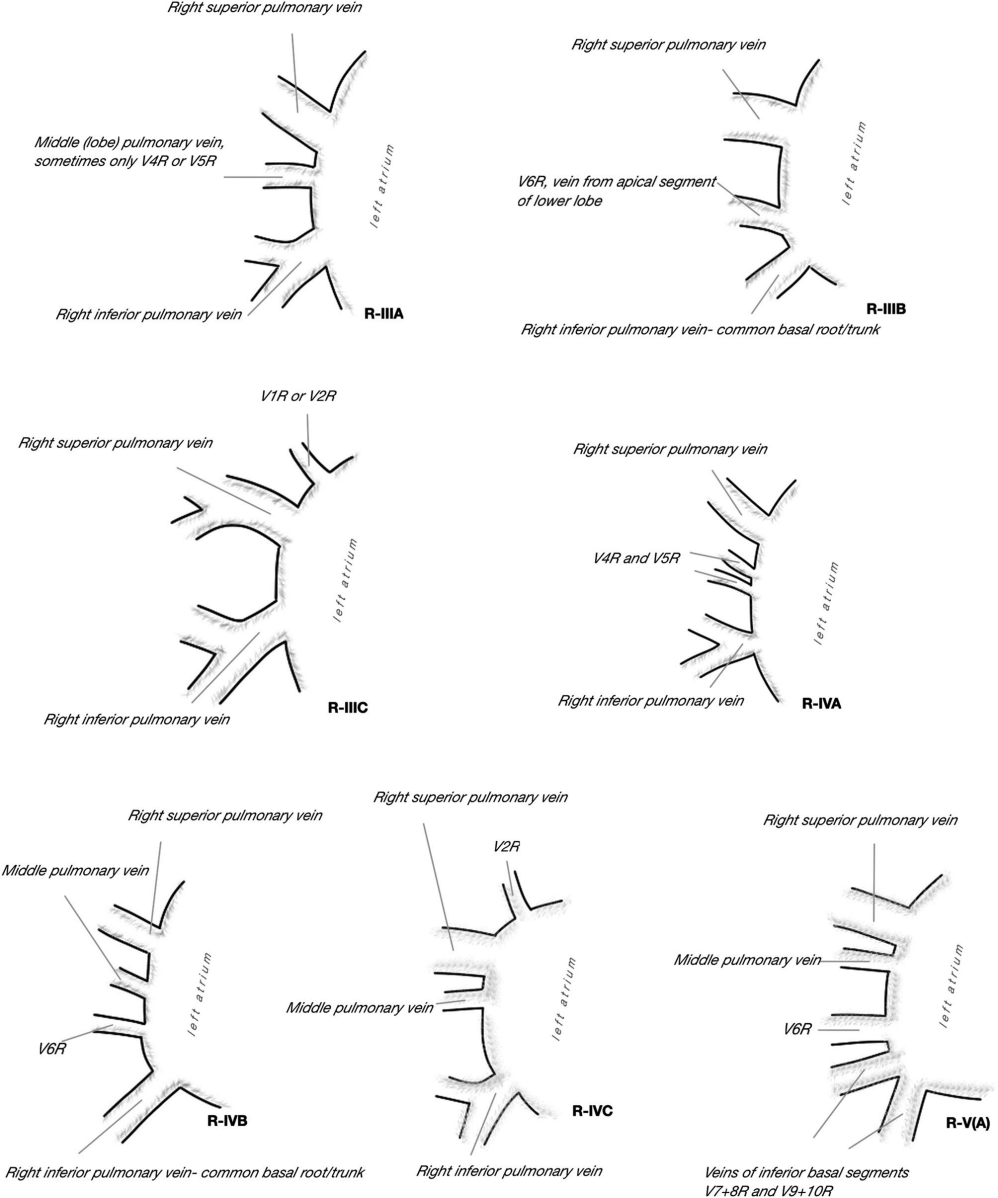
**a, b** Observed patterns of right pulmonary veins outflow into the left atrium

**Fig. 2:**
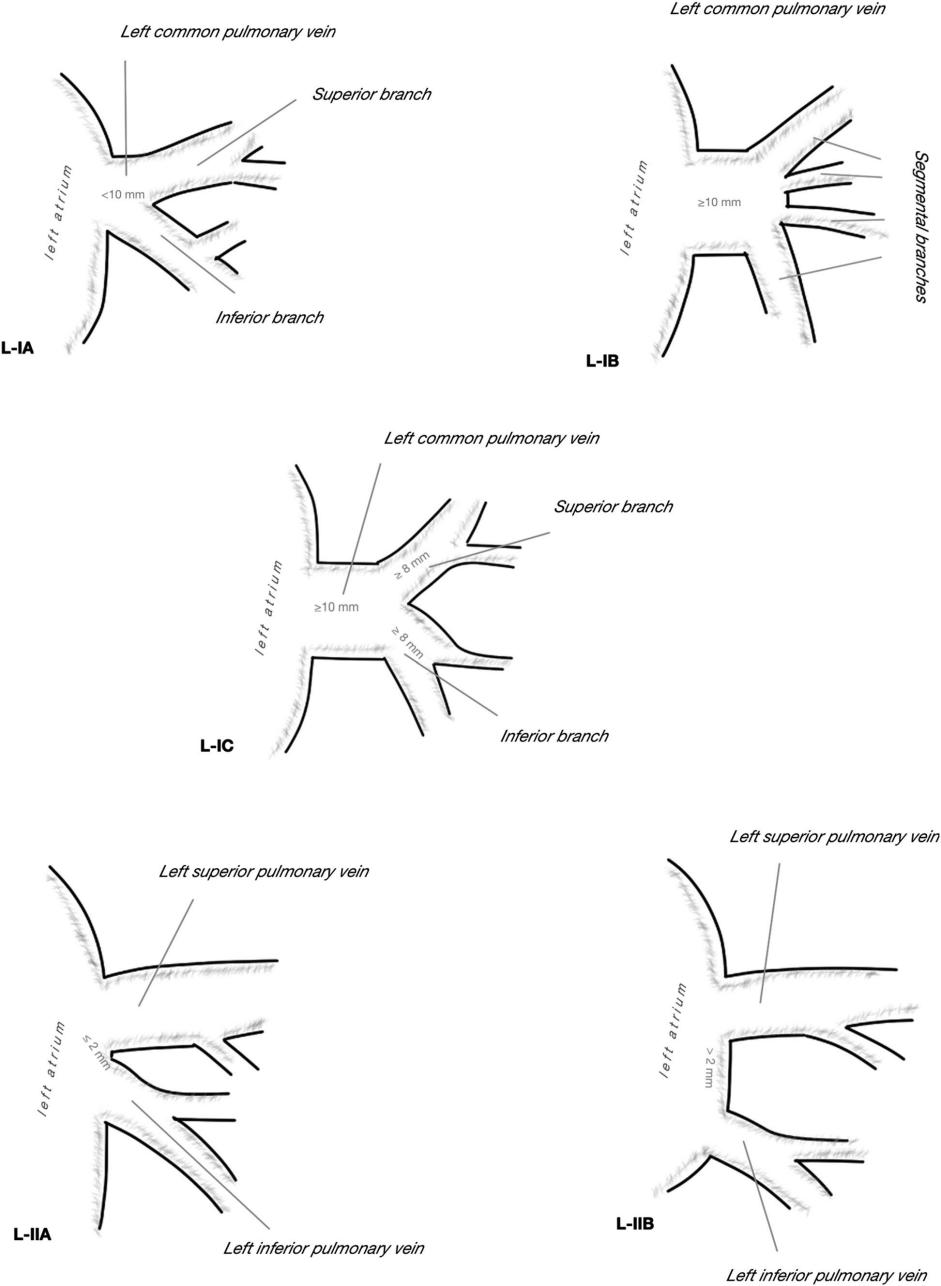
Observed patterns of left pulmonary veins outflow into the left atrium

There was great variability of venous outflow to the LA on the right side, from one to five separate veins were observed, taking into consideration the drainage area and the length of venous trunk, 13 separate drainage patterns were described (Fig. 1). In most cases, there were two separate right pulmonary veins (66.67%), but in 20.74% there was third vein draining the middle lobe or one of its segments. More homogenous venous outflow was observed on the left side, one or two veins emptied into LA and five different drainage patterns were described (Fig. 2). Even if two separate left pulmonary veins were observed in 82.22%, it needs to be acknowledged that in 17.78% a single common trunk of left PVs (commLPV) was found. The frequency of each pattern in relation to sex was not significantly different from the expected: on the right chi^2^ = 12.40, *df* = 25, *p* = 0.98, on the left chi^2^ = 5.76, *df* = 9, *p* = 0.76. In AF+ subgroup, the most frequent type observed on the right side was three right pulmonary veins (a separate vein from the middle lobe) observed in 38.46%. No atypical pattern was proven to be more frequent than expected in AF+ group in comparison to AF*−* group, most likely due to small number of cases in AF+ subgroup. The frequency of all drainage patterns presented in Figs. 1 and 2 is summarized in Table 1.

**Table 1:** Morphometry of proximal pulmonary veins and of veno-atrial ostia

	Mean	Median	Minimal	10th percentile	90th percentile	Maximal	Standard deviation
RSPV, *N* = 129							
RSPV ostium MIN (mm)	11.17	11.30	4.10	7.40	14.50	19.60	2.90
RSPV ostium MAX (mm)	16.46	16.30	7.70	12.40	20.50	22.70	3.00
RSPV ostium narrowing	0.68	0.69	0.30	0.49	0.846	0.95	0.14
RSPV ostium surface area (mm^2^)	150.77	145.90	38.60	78.30	233.10	320.00	57.94
RSPV trunk length (mm)	11.57	10.80	1.70	4.10	19.60	31.50	6.23
MPV, *N* = 34							
MPV ostium MIN (mm)	5.59	5.15	1.60	3.00	7.60	12.30	2.05
MPV ostium MAX (mm)	7.406	7.00	3.80	4.50	10.70	16.80	2.59
MPV ostium surface area (mm^2^)	36.24	27.45	4.90	11.10	64.20	161.60	28.28
MPV trunk length (mm)	10.42	5.75	1.10	3.20	23.10	30.50	8.77
RIPV, *N* = 128							
RIPV ostium MIN (mm)	12.26	12.25	6.50	9.00	15.30	18.90	2.32
RIPV ostium MAX (mm)	15.64	15.50	9.20	12.60	18.90	23.30	2.48
RIPV ostium narrowing	0.79	0.81	0.436	0.60	0.91	0.97	0.11
RIPV ostium surface area (mm^2^)	153.36	147.50	61.00	100.70	210.60	335.00	48.84
RIPV trunk length (mm)	4.87	4.00	0.10	0.90	10.30	13.00	3.43
commRPV, *N* = 6							
commRPV ostium MIN (mm)	16.60	15.35	15.00			22.30	2.856
commRPV ostium MAX (mm)	26.95	26.75	24.60			29.60	2.18
commRPV ostium narrowing	0.615	0.61	0.53			0.75	0.08
commRPV ostium surface area (mm^2^)	358.10	349.60	289.50			514.00	82.34
commRPV trunk length (mm)	6.02	5.20	0.10			14.70	4.89
LSPV, *N* = 111							
LSPV ostium MIN (mm)	11.64	11.50	7.20	8.90	14.20	22.00	2.33
LSPV ostium MAX (mm)	17.40	17.10	12.00	14.30	19.90	32.20	2.86
LSPV ostium narrowing	0.67	0.67	0.43	0.55	0.80	0.89	0.11
LSPV ostium surface area (mm^2^)	163.60	154.50	69.00	108.90	215.60	596.30	62.82
LSPV trunk length (mm)	19.07	18.10	1.80	11.70	26.30	31.00	5.72
LIPV, *N* = 111							
LIPV ostium MIN (mm)	10.18	10.50	3.00	7.00	13.00	17.30	2.365
LIPV ostium MAX (mm)	16.256	16.10	9.90	13.10	19.20	26.20	2.64
LIPV ostium narrowing	0.63	0.64	0.23	0.47	0.81	0.91	0.13
LIPV surface area (mm^2^)	135.59	131.50	56.40	83.80	184.90	294.70	43.68
LIPV trunk length (mm)	12.135	11.90	2.50	7.10	18.40	24.40	4.55
commLPV, *N* = 24							
commLPV ostium MIN (mm)	14.33	13.95	8.90	11.00	18.10	18.70	2.85
commLPV ostium MAX (mm)	26.27	25.90	15.60	20.50	32.00	34.10	4.30
commLPV ostium narrowing	0.55	0.56	0.39	0.42	0.70	0.85	0.12
commLPV ostium surface area (mm^2^)	308.15	295.45	133.30	192.50	439.80	513.50	92.67
commLPV trunk lenght (mm)	14.04	16.60	0.10	3.50	22.20	28.80	8.14

*RSPV* right superior pulmonary vein, *MPV* middle pulmonary vein, *RIPV* right inferior pulmonary vein, *commRVP* common trunk of right pulmonary veins, *LSPV* left superior pulmonary vein, *LIPV* left inferior pulmonary vein, *commLPV* common trunk of left pulmonary veins, *N* number of cases, *MIN* minimal dimension, *MAX* maximal dimension

Detailed morphometry of the most commonly observed pulmonary veins trunks and the openings intto the LA is presented in Table 2. The surface area of left inferior pulmonary vein (LIPV) was smaller than of the three remaining veins, numerically the difference of mean values ranges from 15.18 to 28.01 mm^2^, and the difference was statistically significant, respectively, right superior pulmonary vein (RSPV) vs. LIPV *t* = 2.26, *p* = 0.02, RIPV vs. LIPV *t* = 2.95, *p* = 0.003, left superior pulmonary vein vs. LIPV *t* = 3.86, *p* < 0.001. There was no difference in the surface area of the ostia of RSPV, RIPV and LSPV. There was also no difference between the surface area of the ostium of common trunk of right PVs (commRPV) and commLPV, *t* = 1.20, *p* = 0.24. The narrowing of the ostium decreased (ostium become more round) with the ostium surface increasing, the relation is linear, with mild correlation level, but statistically significant (*r* = 0.115, *p* = 0.01).

**Table 2:** Frequency of different drainage patterns of right and left pulmonary veins to the left atrium with regard of atrial fibrillation; *N* = 135, *N*_(AF−)_ = 122, *N*_(AF+)_ = 13

Dreinage pattern type^a^	Number combined	Frequency combined (%)	Number in AF−	Frequency in AF− (%	Number in AF+	Frequency in AF+ (%)
R-IA	5	3.70	5	4.10%	0	–
R-IB	1	0.74	0	–	1	7.69
R-IIA	7	5.19	5	4.10	2	15.38
R-IIB	6	4.44	5	4.10	1	7.69
R-IIC	38	28.15	37	30.33	1	7.69
R-IID	39	28.89	37	30.33	2	15.38
R-IIIA	28	20.74	23	18.85	5	38.46
R-IIIB	2	1.48	2	1.64	0	–
R-IIIC	3	2.22	3	2.46	0	–
R-IVA	2	1.48	2	1.64	0	–
R-IVB	2	1.48	2	1.64	0	–
R-IVC	1	0.74	1	0.82	0	–
R-VA	1	0.74	0	–	1	7.69
L-IA	8	5.93	6	4.92	2	15.38
L-IB	15	11.11	14	11.48	1	7.69
L-IC	1	0.74	0	–	1	7.69
L-IIA	20	14.81	20	16.39	0	–
L-IIB	91	67.41	82	67.21	9	69.23

*AF−* subgroup without atrial fibrillation, *AF+* subgroup with atrial fibrillation; *N* number of cases

^a^Drainage patterns presented in Figs. 1 and 2

When the median length of the trunks of different veins was compared (RSPV, RIPV, LSPV, LIPV, commLPV) it was found that:

1. the trunk of LSPV is the longest and it is statistically significant (RSPV vs. LSPV: *U* = 2655.50, *Z* = − 8.40, *p* < 0.001; LSPV vs. RIPV: *U* = 273.00, *Z* = 12.84, *p* < 0.001; LSPV vs. LIPV: *U* = 2151.50, *Z* = 8.38, *p* < 0.001; commLPV vs. LSPV: *U* = 887.50, *Z* = − 2.555, *p* = 0.01);
2. the trunk of RIPV is the shortest and the difference is statistically significant (RSPV vs. RIPV: *U* = 2756.00, *Z* = − 9.28, *p* < 0.001; LSPV vs. RIPV: *U* = 273.00, *Z* = 12.84, *p* < 0.001; LIPV vs. RIPV: *U* = 1476.50, *Z* = 10.60, *p* < 0.001; commLPV vs. RIPV: *U* = 554.00, *Z* = 4.98, *p* < 0.001);
3. there is no significant difference in the length of the trunks of RSPV, LIPV and commLPV.

The mean angle between two right PVs was 63.5°, and it ranged from 22° to 110°. If three veins were observing on the right, the angle was evidently wider and the mean value between two peripheral veins was 92.9°. The angle between left PVs was the sharpest mean = 55.7°. The distance between two right pulmonary veins at the ostial level was 5.42 mm (MED = 5.20, SD = 2.37) and in the left it was 4.025 mm (MED = 3.70, SD = 2.46). Even if numerically the difference of the distance is not spectacular (1.4 mm), the distance between right pulmonary veins and left pulmonary veins ostia is statistically different, *t* = 3.23, *p* = 0.0016.

## Discussion

It is well known that age and morphometry of LA are risk factor of AF [15, 20, 33]. LA dimension that is the most important to assess normal morphology is LA sagittal dimension; the mean value in our study (35.00 mm) is consistent with the literature data (28.1–43 mm) [18, 28, 35]. LA_sag_ significantly increases in patients with AF: based upon a sum of 731 literature cases of patients with AF LA_sag_ in AF+ was 39.61 mm, so the proposed arbitrary value of 40.00 mm to predict AF by computed tomography seems reasonable and is also similar with echocardiographic range of normal values of LA_sag_ which is 23–40 mm proposed by Tracz et al. [30]. This norm somehow outdates the 7.0 cm norm of dimension of left atrium in frontal radiograph proposed by Higgins et al. [14]. LA_sag_ was the parameter which has the strongest correlation with age, so the norm would not apply to adolescent and elderly patients.

Despite LIPV being considered by Elliott as the smallest of all PVs [8], there is lack of evidence for this thesis in the literature. There is also no agreement on the biggest of PVs. Merchant et al. [24] proved RSPV to be the greatest and also proved the dominance of right PV vs. the left, in Cronin et al. [6] study RIPV was the greatest. On the other hand, Hamdan et al. [13] showed LSPV to be the biggest PV, what is consistent with our findings. To better understand this confusion some facts should be stated: upper PV is considered longer and wider than lower PV [28] but this observation is true only in supine position. Also dimensions of pulmonary veins ostia (so the surface area as well) rise in AF patients and this is more evident for upper PVs [10, 17, 18, 28]. Good measurement of pulmonary vein ostium is important before planning catheter ablation and after to monitor eventual stenosis of PV [29]. The narrowing of PV is also important feature, our observation that commLPV ostium is the flattest and that RIPV ostium is the roundest are consistent with the literature data [24, 28].

Observed differences of PV trunks length are not only statistically but also clinically important. Short trunk is considered by Stanford and Breen [29] as difficult during catheter ablation and 10 mm was proposed as short by Eliot [8]. In our study, mean length of RIPV trunk was 4.87 mm, and in the literature is 5.4–11 mm, there is no doubt that RIPV has the shortest trunk and in most studies it was half of the length of the rest [1, 5, 6, 21, 28]. In thoracic surgery not only short, but in some cases of long trunk, may pose some risks—there are cases of long trunk of commLPV which was stapled during lobectomy [31] and resulted with serious complications [16]. The mean length of commLPV was in our study 14.04 mm and in the literature ranged 8.1–23.3 mm [1, 17, 21].

The typical anatomy of right and left PV are two separate ostia on each side, this was true in our series in more than 66% on the right and almost 82% on the left, comparing to literature data where the typical anatomy of right PV was observed in 60–88% and of left PV in 41–91% [1, 23, 28, 32]. A common trunk was more frequent on the left (17.8%) than on the right (4%), which is similar to most of the literature data: 6–33% [1, 17, 18, 23, 28] and 0.76–4%, respectively [3, 5, 17, 32].

Separate vein of the middle lobe was observed in more than 25% of cases and was one of three of right PVs in 20.74%, what made it the most common variation of pulmonary vein outflow in our study. This was also observed in the past by other authors in 7.9–26.7% of cases [1, 5, 17, 18, 23, 28, 34]. It is worth mentioning that a separate middle lobe vein was more frequent in AF+ subgroup, in our study in 38% of small number of 13 cases, but a similar observation was made by Marom et al. and it was proven as statistically significant [23]. The less frequent variations of PV outflow pattern are especially important in thoracic surgery, and the good identification is key to avoid surgical complications [2, 4].

Morphological features of pulmonary veins and morphometry of the left atrium and pulmonary veins are important for clinical purposes and our results are accordance with previous papers. With the current data, the range of normal values and typical drainage patterns can help to better identify potentially risky variations and to better prepare for surgery or catheter ablation.
